# Fuyuan Decoction Enhances SOX9 and COL2A1 Expression and Smad2/3 Phosphorylation in IL-1*β*-Activated Chondrocytes

**DOI:** 10.1155/2015/821947

**Published:** 2015-12-07

**Authors:** Yudi Zhang, Rongheng Li, Yu Zhong, Sihan Zhang, Lingyun Zhou, Shike Shang

**Affiliations:** ^1^Department of Combination of Chinese and Western Medicine, The First Affiliated Hospital of Chongqing Medical University, Chongqing 400016, China; ^2^College of Laboratory Medicine, Chongqing Medical University, Yuzhong District, Chongqing 400016, China; ^3^Longgang District People's Hospital of Shenzhen, Longgang District, Shenzhen, Guangdong 518172, China

## Abstract

Fuyuan Decoction (FYD), a herbal formula in China, has been widely used for osteoarthritis (OA) treatment. Herein, we determined the effects of FYD on the expression of transcription factor SOX9 and its target gene collagen type II, alpha 1 (COL2A1) as well as the activation of Smad2/3 in interleukin- (IL-) 1*β*-stimulated SW1353 chondrosarcoma cells. Serum-derived FYD (FYD-CS) was prepared to treat SW1353 cells with or without SB431542, a TGF-*β*1 receptor inhibitor. Cell cycle progression was tested by flow cytometry. The expression of SOX9 and COL2A1 and the activation of Smad2/3 (p-Smad2/3) were analyzed by quantitative reverse transcription polymerase chain reaction (qRT-PCR) and/or western blot. The results showed that, after treatment, FYD-CS, while inducing S-phase cell cycle arrest, enhanced cell proliferation and protected the cells against IL-1*β*- and/or SB431542-induced cell growth inhibition. Furthermore, FYD-CS reversed the decreased expression of COL2A1 and SOX9 induced by IL-1*β* and SB431542 and blocked the decreased phosphorylation of Smad2/3 induced by IL-1*β* alone or in combination with SB431542. Our results suggest that FYD promotes COL2A1 and SOX9 expression as well as Smad2/3 activation in IL-1*β*-induced chondrocytes, thus benefiting cell survival.

## 1. Introduction

Fuyuan Decoction (FYD) is an empirical formula used to treat Bi Zheng in clinical practice and has been proven effective in the treatment of osteoarthritis (OA). FYD can inhibit the development of OA [1]. To date, several bioactive components of FYD have been identified, including icariin and arasaponin R1, which exhibit a number of biological activities, including inhibiting inflammation and oxidative damage [2] as well as the development and progression of OA [3]. In addition, pilose antler, another component of FYD, has been found to increase the expression of Smad2/3 in OA cartilage [4].

In the early stages of OA, the inflammatory response is an important contributing factor that initiates and promotes the disease. Several inflammatory mediators have been identified to be responsible for the inflammatory pathology of OA [5–7]; among them, interleukin-1 beta (IL-1*β*) plays a key role in amplifying the inflammatory response and, in combination with dysregulated other factors including transforming growth factor-*β* (TGF-*β*) [8], exacerbates the pathogenesis of OA [9]. TGF-*β* is a multifunctional growth factor that plays an important role in the formation, homeostasis, and repair of cartilage [10–12]. TGF-*β* stimulates the formation of cartilage, including chondrogenic condensation [13, 14] and chondroprogenitor cell proliferation and differentiation [15, 16]. It also inhibits the terminal differentiation of chondrocytes into the hypertrophic phenotype by intercepting the calcification of cartilage matrix, differentiation, and ossification of osteoblasts [12, 17], resulting in the formation of articular cartilage at the end of long bones [18].

Of the current treatment options for OA, nonsteroidal anti-inflammatory drugs and selective cyclooxygenase-2 inhibitors are widely used. However, due to the potential side effects and concerned effectiveness of these agents [19–21], it is necessary to develop more safe and effective alternative drugs for the treatment of OA. In the context of traditional Chinese medicine, OA belongs to the category Bi Zheng, which is defined as a syndrome marked by arthralgia and dyskinesia of the joints and limbs due to the meridians of the limbs being attacked by wind, dampness, and heat or cold pathogens. FYD is an empirical formula used to treat Bi Zheng in clinical practice and has been proven effective in the treatment of OA. Now, Fuyuan capsule made from FYD has been confirmed to have a better effect on the treatment of experimental OA than the positive control western medicine glucosamine hydrochloride [2]. The purpose of this study was to investigate the effects and mechanisms of FYD in chondrocytes in the OA microenvironment. In particular, the expression levels of SOX9 and COL2A1 as well as the phosphorylation of Smad2/3 were analyzed.

## 2. Materials and Methods

### 2.1. Materials and Reagents

Notoginsenoside R1, icariin, and digoxin were purchased from the National Institute for the Control of Pharmaceutical and Biological Products (Beijing, China). SB431542, a TGF-*β*1 receptor inhibitor, was obtained from Selleckchem (Houston, TX, USA). IL-1*β* was purchased from PeproTech (Rocky Hill, NJ, USA). Cell Counting Kit-8 (CCK-8) was obtained from Beyotime Biotechnology (Shanghai, China). The mRNA primers were synthesized by Sangon Biotech (Shanghai, China). TRIzol reagent was obtained from Ambion (Grand Island, NY, USA). The RevertAid First Strand cDNA Synthesis Kit was obtained from Thermo Fisher Scientific Inc. (Waltham, MA, USA). The SYBR Green PCR Master Mix Kit was provided by the College of Laboratory Medicine, CWBIO (Beijing, China). Rabbit anti-GAPDH antibody was obtained from Zhongding (Nanjing, China). Rabbit anti-P-Smad2/Smad3 antibodies were obtained from Cell Signaling Technology (Danvers, MA, USA). COL2A1 and SOX9 antibodies were obtained from Bioworld Technology (Minneapolis, MN, USA). Horseradish peroxidase- (HRP-) conjugated goat anti-rabbit secondary antibody was obtained from ABGENT (San Diego, CA, USA). Western blot Chemiluminescent HRP was provided by the College of Laboratory Medicine (Immobilon Western, USA).

### 2.2. Herbal Preparation

**Figure 1:**
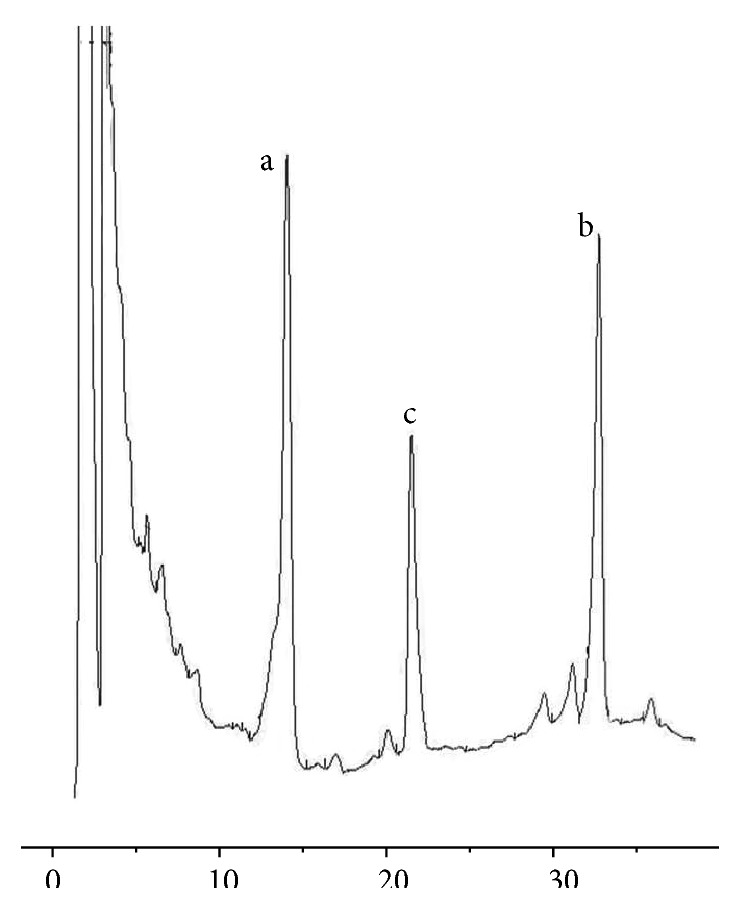
HPLC chromatograms of notoginsenoside R1 and icariin in FYD. a: notoginsenoside R1; b: icariin; and c: digoxin as an internal standard.

FYD was prepared from nine dried powdered plant species as follows: 15 g of* Epimedium brevicornum*, 15 g of* Astragalus membranaceus*, 15 g of* Davallia formosana*, 15 g of* Psoralea corylifolia*, 10 g of* Angelica sinensis*, 10 g of* Panax ginseng*, 5 g of* Panax pseudoginseng* var.* notoginseng*, 10 g of* Salvia miltiorrhiza*, and 5 g of* Glycyrrhiza uralensis*. These plant materials were from Chongqing Tongjunge Pharmacy (Chongqing, China) and identified by Professor Rongheng Li, The First Affiliated Hospital of Chongqing Medical University (Chongqing, China). Currently, these dried powdered plant species are being stored in the Department of Pharmacy of The First Affiliated Hospital of Chongqing Medical University. FYD was extracted according to the standard methods recommended by the Chinese Pharmacopoeia (2010). In short, the herbal mixture was extracted twice in boiling water for 2 h each, and the final residues were filtered using a 0.45 *μ*m microfilter, concentrated, and then made into a freeze-dried powder (1). The extraction yield of FYD was 13.67% (w/w), containing 12.18 mg of notoginsenoside R1 and 54.65 mg of icariin per g of freeze-dried powder, according to a high-performance liquid chromatography (HPLC) method (Figure 1). The resultant powder was subsequently dissolved in sterile water at the desired concentrations for the animal studies.

### 2.3. Preparation of FYD-Containing Serum (FYD-CS)

Four-month-old New Zealand rabbits weighing 1800–2000 g were purchased from the Chongqing Medical Laboratory Animal Center (License, SYXK (yu) 2012-0001). Animal care and all experimental procedures were approved by the Ethics Committee of Animal Research of Chongqing University of Medical Sciences (CUMS11-66). Rabbits were randomly assigned to two groups containing two animals each. One group was orally administered with 4.07 g/kg/d FYD (5.8 mL/rabbit) twice daily for 7 consecutive days, while the other group was gavaged with an equal volume of physiological saline. This dose and regimen were picked based on extrapolation of the dosage for humans used in our clinical practice. Approximately 2 h after the last administration on day 7, the rabbits were euthanized by injection of pentobarbital sodium at a dose of 40 mg/kg in the marginal ear vein, and blood was retrieved from the carotid artery. Serum from both groups (FYD-CS and Con-s) was collected by centrifugation at 3000 ×g for 20 min at 4°C, then filtered through a 0.22 *μ*m filter, and stored in −20°C in aliquots.

### 2.4. SW1353 Cell Culture and Treatment

Human chondrosarcoma cells (SW1353) have a similar phenotype as chondrocytes [22, 23]; therefore, they were used instead of cartilage cells to evaluate the regulatory effects of FYD in this study. The SW1353 cells were obtained from the Institute of Biochemistry and Cell Biology (Shanghai, China). The cells were grown in Dulbecco's modified Eagle's medium supplemented with 10% (v/v) fetal bovine serum, 100 IU/mL penicillin, 100 *μ*g/mL streptomycin, and 2 mM glutamine at 37°C in a 5% CO_2_ incubator. When the cells reached 85% confluence, different concentrations of FYD-CS (5–25% (v/v)) and/or 10 *μ*M SB431542 were added. One hour later, 10 ng/mL IL-1*β* was added to stimulate the cells. The cells were further incubated for 24–96 h before assaying cell viability, gene expression, and protein expression.

### 2.5. Cell Viability

SW1353 cells were incubated in 96-well plates (0.5 × 10^4^ cells/well) in the presence or absence of the indicated concentrations of FYD-CS, Con-s, SB431542, and/or IL-1*β* for 24–96 h. Next, 20 *μ*L of CCK-8 was added to each well, and the plates continued to be incubated at 37°C for an additional 4 h. The optical density values were measured at 450 nm on a microplate reader.

### 2.6. Cell Cycle Assay

SW1353 cells were grown in 6-well plates (2 × 10^5^ cells per well) in the presence or absence of the indicated concentrations of FYD-CS, Con-s, SB431542, and/or IL-1*β*. After treatment for 72 h, the cells were harvested and washed three times with cold phosphate-buffered saline (PBS) and then fixed in cold 75% ethanol for at least 8 h at 4°C. After fixation, the cells were treated with PI/RNase staining followed by fluorescence-activated cell sorting analysis.

### 2.7. Quantitative Reverse Transcription Polymerase Chain Reaction (RT-qPCR)

Total RNA was extracted using TRIzol reagent as recommended by the manufacturer. First-strand cDNA synthesis was performed using the Thermo Scientific RevertAid First Strand cDNA Synthesis Kit. The SYBR Green PCR Master Mix Kit was used for real-time PCR to determine the relative quantification of mRNA. *β*-actin was used as an internal control. The following primer pairs were used: *β*-actin (XM_006715764.1), 5′-AAAGACCTGTACGCCAACAC-3′ (forward) and 5′-GTCATACTCCTGCTTGCTGAT-3′ (reverse); COL2A1 (XM_006719242.1), 5′-AACCAGATTGAGAGCATCCG-3′ (forward) and 5′-AACGTTTGCTGGATTGGGGT-3′ (reverse); and SOX9 (NM_000346.3), 5′-GCTCTGGAGACTTCTGAACGA-3′ (forward) and 5′-CCGTTCTTCACCGACTTCCT-3′ (reverse). The PCR primers were designed by Invitrogen Biotechnology (Shanghai, China). Quantitative (real-time) PCR was performed using a Bio-Rad CFX96 Real-Time PCR System (Hercules, CA, USA) with 40 cycles of 95°C for 10 min, 95°C for 10 s, and 58°C for 30 s; measurements were made at the end of a 58°C annealing step. Data were analyzed using Bio-Rad CFX Manager software (version 2.0). The 2^−ΔΔCT^ method was used to calculate the relative fold changes of the COL2A1 and SOX9 mRNA expression.

### 2.8. Western Blot Analysis

The SW1353 cells were washed three times with ice-cold PBS and lysed with cell lysis buffer (20 mM Tris-HCl (pH 7.5), 150 mM NaCl, 1% EDTA, 1% TritonX-100, and 2.5 mM sodium pyrophosphate) supplemented with 1 mM phenylmethylsulfonyl fluoride, 1 mM NaF, and 1 mM sodium orthovanadate. Equal amounts of cellular protein (40 *μ*g per well) were separated on a 12% sodium dodecyl sulfate-polyacrylamide gel and electrophoretically transferred to a nitrocellulose membrane. After blocking with 5% fat-free milk and 0.1% Tween 20 in PBS, the membrane was incubated with a primary antibody (anti-GAPDH (1 : 1000), anti-p-Smad2/Smad3 (1 : 1000), anti-COL2A1 (1 : 500), or anti-SOX9 (1 : 500)) overnight at 4°C and then a HRP-conjugated goat anti-rabbit secondary antibody for 2 h at room temperature. The immunocomplexes were visualized using a chemiluminescent HRP substrate, and band intensities were quantitated using densitometry and normalized to the density of GAPDH from the same treatment group.

### 2.9. Statistical Analysis

All experiments were performed in triplicate using independent samples. The data were presented as means ± standard deviation (SD) and analyzed using SPSS (version 19.0, SPSS Inc., Chicago, IL, USA). Differences between groups were analyzed by analysis of variance, and *P* < 0.05 was considered statistically significant.

## 3. Results

### 3.1. FYD-CS Enhances Cell Viability

**Figure 2:**
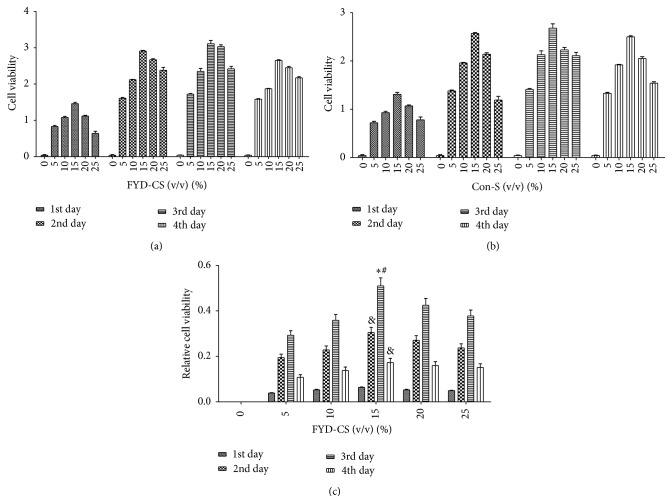
Effect of FYD-CS on cell viability in IL-1*β*-activated SW1353 cells. The cells were treated with different concentrations of FYD-CS or Con-s for 24–96 h; then cell viability was assayed by a colorimetric kit. The data are expressed as the mean ± SD (*n* = 3). (a) Cells treated with different concentrations of FYD-CS. (b) Cells treated with the corresponding concentration of blank serum as used in the FYD-CS treatments. (c) The effect of FYD alone, which was obtained by subtracting the blank serum results from the FYD-CS results. ^*∗*^ *P* < 0.05 versus 10% FYD-CS, ^#^ *P* < 0.05 versus 15% FYD-CS, and ^&^ *P* < 0.05 versus 15% FYD-CS on day 3.

To determine the cytotoxicity and optimal dose of FYD-CS, IL-1*β*-activated SW1353 cells were treated with different concentrations of FYD-CS for different periods of time. As shown in Figure 2, 5–15% FYD-CS stimulated cell proliferation in a concentration-dependent manner. However, higher concentrations of FYD-CS reduced cell viability when compared to 15% FYD-CS, suggesting that 15% FYD-CS achieved the maximal enhancing effect of cell viability in IL-1*β*-induced SW1353 cells, especially at 72 h after treatment. Therefore, this concentration was used in the subsequent experiments.

**Figure 3:**
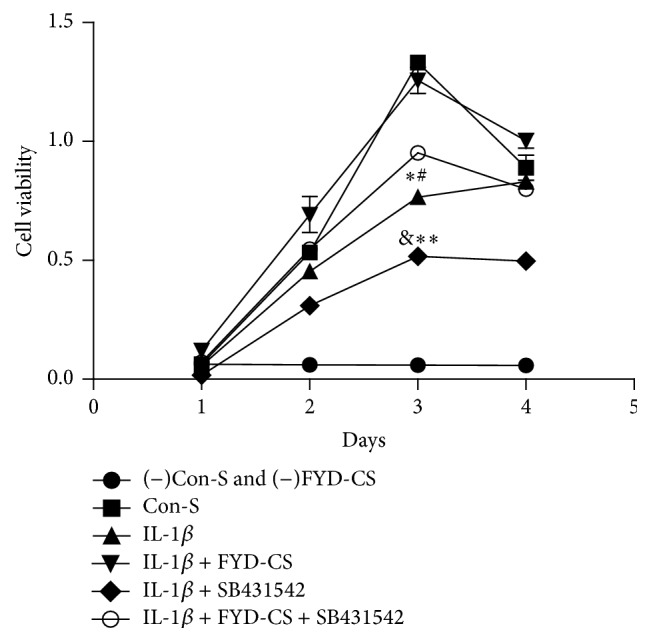
Effect of FYD-CS on cell viability in IL-1*β*- and/or SB431542-treated SW1353 cells. The cells were treated with 15% FYD-CS, 10 *μ*M SB431542, and/or 10 ng/mL IL-1*β* for 24–96 h, and then the cell viability was assayed. The data are expressed as the mean ± SD (*n* = 3). We found that cell growth peaked at 72 h. ^*∗*^ *P* < 0.05 versus the control; ^#^ *P* < 0.05 versus the group treated with both IL-1*β* and FYD-CS; ^&^ *P* < 0.05 versus the group treated with IL-1*β* alone; ^*∗∗*^ *P* < 0.05 versus the group treated with IL-1*β*, FYD-CS, and SB431542.

**Figure 4:**
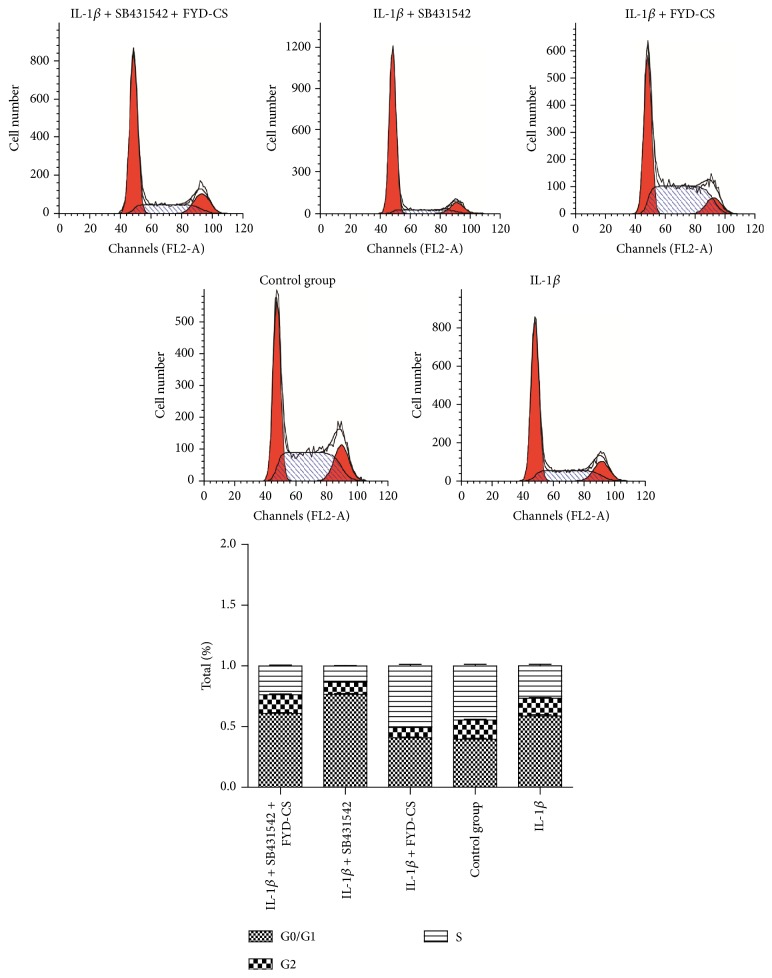
Effect of FYD-CS on cell cycle progression in IL-1*β*- and/or SB431542-treated SW1353 cells. The cells were treated with 15% FYD-CS, 10 *μ*M SB431542, and/or 10 ng/mL IL-1*β* for 72 h, and then cell cycle progression was assayed.

In another set of experiments, the involvement of TGF-1*β* signaling in the FYD-CS regulation of cell growth was evaluated. As shown in Figures 3 and 4, IL-1*β* induction reduced SW1353 cell proliferation, inducing cell cycle G0/G1 arrest, compared to the Con-s control. When IL-1*β*-activated cells were treated with 15% FYD-CS and/or 10 *μ*M SB431542 for 72 h, FYD-CS completely reversed the IL-1*β*-mediated inhibition of cell viability, inducing cell cycle arrest at the S phase, while the ALK5 inhibitor enhanced the IL-1*β*-mediated inhibition. Interestingly, FYD-CS completely abolished the inhibitory effect of the ALK5 inhibitor, indicating that chondrocyte growth needs the help of the TGF-1*β* receptor and TGF-1*β* signaling and that FYD-CS protects SW1353 cells against ALK5 inhibitor-induced inhibition of IL-1*β*-activated cells.

### 3.2. FYD-CS Upregulates the Gene Levels of COL2A1 and SOX9

**Figure 5:**
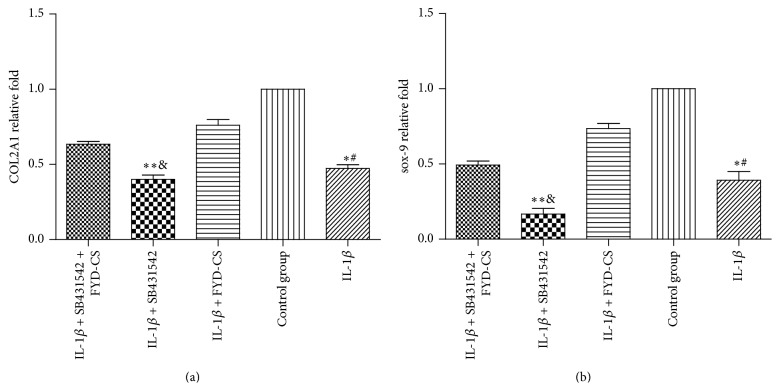
Quantitative RT-PCR assay of COL2A1 and SOX9 in IL-1*β*-activated SW1353 cells pretreated with 15% FYD-CS and/or 10 *μ*M SB431542. The data are expressed as the mean ± SD (*n* = 3). ^*∗*^ *P* < 0.05 versus the control. ^#^ *P* < 0.05 versus the group treated with IL-1*β* and FYD-CS; ^*∗∗*^ *P* < 0.05 versus the group treated with IL-1*β*; ^&^ *P* < 0.05 versus the group treated with IL-1*β*, FYD-CS, and SB431542.

To further understand the underlying mechanisms of FYD-CS in the regulation of IL-1*β*- and SB431542-induced cell proliferation inhibition, the mRNA expression levels of COL2A1 and SOX9 were detected in IL-1*β*-activated cells treated with 15% FYD-CS and/or SB431542 by qRT-PCR (Figure 5). The results showed that FYD-CS significantly recovered the IL-1*β*-induced decrease in the gene expression of COL2A1 and SOX9. Moreover, SB431542 and IL-1*β* showed a synergistic inhibitory effect on the expression of the two genes, but this inhibition was partially blocked by FYD-CS. These data suggest that FYD-CS promotes collagen type II expression in IL-1*β*-activated SW1353 cells, partially through the classical TGF-*β* signaling pathway.

### 3.3. FYD-CS Enhances Smad2/Smad3 Phosphorylation as well as COL2A1 and SOX9 Protein Expression

**Figure 6:**
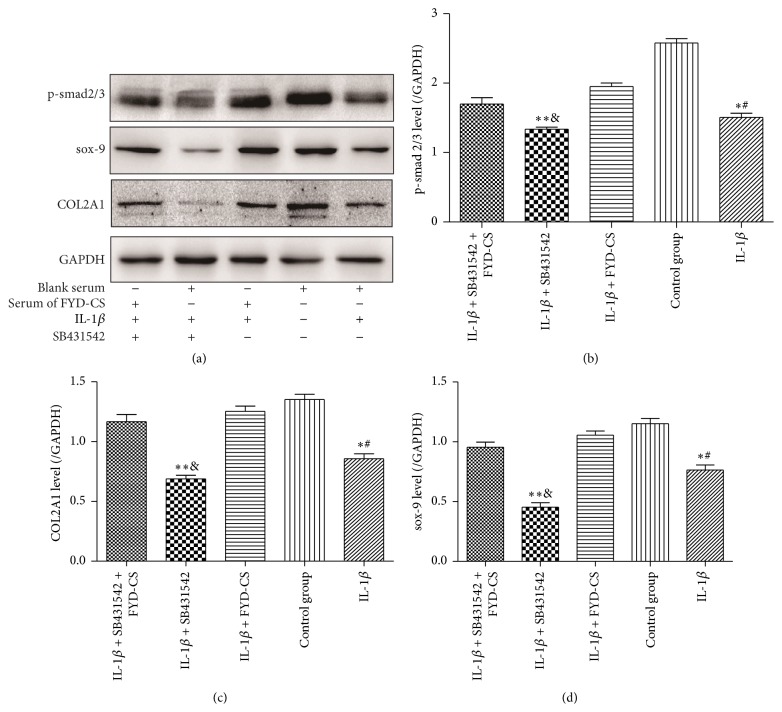
Western blot assay of COL2A1, SOX9, and p-Smad2/3 in IL-1*β*-activated SW1353 cells pretreated with 15% FYD-CS and/or 10 *μ*M SB431542. Representative bands are shown. The band intensities were compared to that of the corresponding GAPDH band with the same treatment. The data are expressed as the mean ± SD (*n* = 3). ^*∗*^ *P* < 0.05 versus the control; ^#^ *P* < 0.05 versus the group treated with IL-1*β* and FYD-CS; ^*∗∗*^ *P* < 0.05 versus the group treated with IL-1*β*; ^&^ *P* < 0.05 versus the group treated with IL-1*β*, FYD-CS, and SB431542.

To examine the effects of FYD-CS on the protein expression of COL2A1 and SOX9 as well as the activation of TGF-1*β* signaling, IL-1*β*-activated cells were treated with 15% FYD-CS and/or SB431542 for 72 h, and the expression levels of COL2A1, SOX9, and p-Smad2/Smad3 were analyzed by western blot (Figure 6). It was found that the expression levels of p-Smad2/Smad3, COL2A1, and SOX9 were significantly decreased in IL-1*β*-treated cells when compared to the baseline SW1353 cells (*P* < 0.05); however, FYD-CS cotreatment abrogated the effect of IL-1*β* (*P* < 0.05). Moreover, SB431542 further decreased the IL-1*β*-induced inhibition of COL2A1 and SOX9 protein expression and Smad2/Smad3 phosphorylation, but this synergistic effect on TGF-1*β* signaling and collagen type II synthesis was significantly suppressed by FYD-CS (*P* < 0.05). These data further confirmed that FYD-CS regulates IL-1*β*-mediated Smad2/3 phosphorylation as well as COL2A1 and SOX9 expression, which were partially dependent on the classical TGF-*β* signaling pathway.

## 4. Discussion

OA is a common chronic degenerative joint disease in the elderly. The typical pathological changes of OA include articular cartilage impairment, bone sclerosis, osteophytes, and subchondral synovial tissue fibrosis. Although the pathogenesis of OA remains obscure, it is generally attributable to the interactions of multiple factors including mechanical and biological factors that cause an imbalance between tissue damage and repair and resultant degradation of joint tissues, especially articular cartilage.

Chondrocytes are the only cells found in cartilage and are embedded in an extensive extracellular matrix (ECM) [24]. Collagens and proteoglycans are the main components of the ECM [24]. The majority of the collagen in articular cartilage is type II collagen, which provides the tissue with tensile strength [25, 26]. The major proteoglycan of articular cartilage is aggrecan, which provides structural support by retaining water in the matrix [27]. SOX9 is a transcription factor essential for regulating the expression of many cartilage ECM genes, including COL2A1 [28].

TGF-*β* signaling functions through cell surface heterogenic receptor complexes containing type II and type I (ALK5 or ALK1) receptors to phosphorylate two receptor-regulated Smad proteins, Smad2 and Smad3. Smad2/3 phosphorylation mediates anabolic signaling in chondrocytes essential for articular cartilage matrix turnover and homeostasis [10, 12]. The TGF-*β*/Smad2/3 signaling pathway is truncated by using a powerful combination of competitive ALK5 receptor inhibitors. Therefore, these inhibitors can affect the synthesis of downstream target genes. This combination is very strong, so the use of these inhibitors can simulate the reduced expression of TGF-*β* receptors to the maximum limit* in vitro* [29].

In normal chondrocytes, TGF-*β* signals the phosphorylation of Smad2/3 predominantly via ALK5, resulting in the formation of a complex with Smad4 and translocation into the nucleus, where it regulates the expression of target genes such as type II collagen and aggrecan. However, in OA, TGF-*β* may phosphorylate Smad1 and Smad5 preferentially via ALK1, leading to increased expression of matrix metalloproteinase 13 and decreased expression of type II collagen and aggrecan. It has been shown that the phosphorylated Smad2 level is reduced in cartilage during the progression of OA in both spontaneous and collagenase-induced OA mouse models [30]. Also, Smad2 phosphorylation in cartilage of old mice is lower than that of young mice [31]. Another study has reported that the decreased levels of phosphorylated Smad3 in Smurf-2 transgenic mice cause the development of an OA-like phenotype [32]. Additionally, Smad3 knockout mice develop a degenerative joint disease resembling human OA [18] and intervertebral disc degeneration [33]. Moreover, some genetic studies have shown that Smad3 gene mutations are a risk factor for the susceptibility to OA. Therefore, the expression levels of TGF-*β* receptors and their downstream proteins Smad2/3 are intimately associated with OA pathogenesis.

In the present study, we used serum-derived FYD as the drug source to mirror the* in vivo* biotransformation and pharmacological effects of FYD and to minimize other uncertain confounding factors [34, 35]. We focused on TGF-*β* signaling and SOX9 expression because they have a pivotal role in the pathophysiology of joint cartilage. One of the aims of this study was to simulate the TGF-*β* receptor expression level decrease by using receptor inhibitors. When the receptors were completely inhibited, the expression of phosphorylated Smad2/3 in chondrocytes was further downregulated, thus achieving the purposes of the experimental modelling. FYD could still activate certain Smad2/3 protein phosphorylation in the presence of SB431542, but the level was lower than that in the no SB431542 inhibitor group. These results indicated that FYD could activate Smad2/3 protein phosphorylation through the TGF-*β* receptor as well as by other means.

We demonstrated that the water-soluble active components of FYD-CS increase Smad2/3 phosphorylation and promote COL2A1 and SOX9 expression, leading to increased cell proliferation in IL-1*β*-induced SW1353 cells. This regulatory process may partly depend on the classical TGF-*β* signaling pathway.

Healthy articular cartilage depends on the balance between anabolic and catabolic cytokines and growth factors. Accumulated evidence supports the association of OA with reduced TGF-*β*/ALK5/Smad2/3 signaling. IL-1*β*, a proinflammatory cytokine and a critical catabolic factor, has been found recently to induce MMP production, reduce Smad2/3 phosphorylation, and inhibit Smad3/4 activity and DNA binding [9]. In the current study, IL-1*β* was used to establish a cellular OA model in SW1353 cells [36]. We found a rapid decrease in the expression levels of p-Smad2/3, COL2A1, and SOX9 in response to IL-1*β*; however, FYD-CS effectively abolished the inhibitory effects of IL-1*β* in SW1353 cells. To the best of our knowledge, this is the first study to determine that FYD-CS is a potent activator of Smad2/3 activity. Moreover, we found that SB431542, an inhibitor of the TGF-*β*1 receptor, not only reduced the levels of phosphorylated Smad2/3 but also reduced the expression levels of COL2A1 and SOX9. In addition, FYD-CS antagonized the effects of the inhibitor, further confirming the role of FYD-CS in the TGF-*β* signaling pathway.

FYD-CS is clinically effective in the treatment of OA. In this study, we explored the mechanism of FYD-CS in regulating the expression of COL2A1 and SOX9 in IL-1*β*-induced SW1353 cells. The results suggest that FYD-CS increases the levels of phosphorylated Smad2/3 as well as the expression of COL2A1 and SOX9 in IL-1*β*-induced SW1353 cells.

## 5. Conclusion

In summary, our data support FYD as a beneficial agent in the treatment of OA; it stimulates cell proliferation, possibly through the positive regulation of TGF-*β* signaling and its target genes that participate in the structure and function of articular cartilage. These noteworthy findings will help to treat OA by repairing the ECM. However, further studies are needed to confirm the regulation of the TGF-*β*/Smad2/Smad3 signaling pathway by FYD in animal models.
